# Refer‐MCI: Enriching referrals of patients to memory clinics for mild cognitive impairment (MCI)

**DOI:** 10.1002/alz70857_106521

**Published:** 2025-12-26

**Authors:** Emrah Düzel, Jochen René Thyrian, Hannes Vossfeld, David Berron, Frank Jessen, Annika Spottke, Stefan Teipel

**Affiliations:** ^1^ German Center for Neurodegenerative Diseases (DZNE), Magdeburg, Germany; ^2^ Institute of Cognitive Neurology and Dementia Research (IKND), Otto‐von‐Guericke University, Magdeburg, Germany; ^3^ Institute of Cognitive Neuroscience, University College London (UCL), London, United Kingdom; ^4^ German center for neurodegenerative diseases (DZNE), Greifswald, Germany; ^5^ Neotiv GmbH, Magdeburg, Germany; ^6^ Deutsches Zentrum für Neurodegenerative Erkrankungen e. V. (DZNE) Bonn, Bonn, Germany; ^7^ Department of Psychiatry, Medical Faculty, University of Cologne, Cologne, Germany; ^8^ German Center for Neurodegenerative Diseases (DZNE), Bonn, Germany; ^9^ German Center for Neurodegenerative Diseases, Rostock, Germany

## Abstract

**Background:**

A timely diagnosis of mild cognitive impairment (MCI) in Alzheimer's disease (AD) is crucial for early interventions, but its broad implementation in health‐care is often challenging due to the complexity and time burden of required cognitive assessments. In the current German health care system, less than 10% of people with a clinical syndrome of MCI are being identified in primary care, even if specialists are included in the assessment. To address these challenges, the usability of new unsupervised digital remote assessment tools needs to be validated in a routine care context.

**Method:**

The Refer‐MCI study will compare diagnosis and referral decisions based on a remote digital self‐assessment (neotivCare app) with usual‐care based referral decision established in participating office‐based specialist care. The reference test will be the diagnosis of MCI due to AD at memory clinics according to S3 guidelines. All patients will also undergo an office‐based MoCA assessment. Blood‐based biomarkers for AD (pTau‐217) will be obtained at memory clinics.

**Result:**

*N* = 500 office‐based care (*N* = 27 offices) patients, aged 60‐80 years who seek advice for self‐reported or carer‐reported cognitive complaints will be screened for participation, 75% (*n* = 400) are expected to consent. For all patients, the office‐based outpatient specialists will document their diagnosis and referral decision prior to and following the use of neotivCare. First patient in is expected in May 2025 and last patient out in February 2026.

**Conclusion:**

The goal of Refer‐MCI is to investigate the potential of digital remote home‐based assessment for improving efficient and timely referral of patients who are likely to have MCI from office‐based specialized care to memory clinics. Refer‐MCI will assess whether remote digital self‐assessment of patients with memory complaints at office‐based specialist care can enrich referrals to memory clinics with MCI. Refer‐MCI will identify barriers for implementing tools for efficient and timely referral of these patients. Established memory clinic‐based testing and blood‐based biomarkers will be used as reference standard to estimate the proportion of MCI due AD among the referred patients.

